# Weighted gene co-expression network analysis reveals key module and hub genes associated with the anthocyanin biosynthesis in maize pericarp

**DOI:** 10.3389/fpls.2022.1013412

**Published:** 2022-10-31

**Authors:** Tingchun Li, Yiting Wang, Qing Dong, Fang Wang, Fanna Kong, Guihu Liu, Yanli Lei, Huaying Yang, Yingbing Zhou, Cheng Li

**Affiliations:** ^1^ Tobacco Research Institute, Anhui Academy of Agricultural Sciences, Hefei, China; ^2^ School of Life Sciences, Anhui Agricultural University, Hefei, China

**Keywords:** anthocyanins, pericarp, comparative dynamic transcriptome analysis, weighted gene co-expression network analysis, maize

## Abstract

Anthocyanins are the visual pigments that present most of the colors in plants. Its biosynthesis requires the coordinated expression of structural genes and regulatory genes. Pericarps are the rich sources of anthocyanins in maize seeds. In the experiment, the transcriptomes of transparent and anthocyanins-enriched pericarps at 15, 20, and 25 DAP were obtained. The results output 110.007 million raw reads and 51407 genes’ expression matrix. Using data filtration in R language, 2057 genes were eventually identified for weighted gene co-expression network analysis. The results showed that 2057 genes were classified into ten modules. The cyan module containing 183 genes was confirmed to be the key module with the highest correlation value of 0.98 to the anthocyanins trait. Among 183 genes, seven structural genes were mapped the flavonoid biosynthesis pathway, and a transcription factor *Lc* gene was annotated as an anthocyanin regulatory gene. Cluster heatmap and gene network analysis further demonstrated that *Naringenin, 2-oxoglutarate 3-dioxygenase* (*Zm00001d001960*), *Dihydroflavonol 4-reductase* (*Zm00001d044122*), *Leucoanthocyanidin dioxygenase* (*Zm00001d014914*), anthocyanin regulatory *Lc* gene (*Zm00001d026147*), and *Chalcone synthase C2* (*Zm00001d052673*) participated in the anthocyanins biosynthesis. And the transcription factor anthocyanin regulatory *Lc* gene Zm00001d026147 may act on the genes *Chalcone synthase C2* (*Zm00001d052673*) and *Dihydroflavonol 4-reductase* (*Zm00001d044122*). The yeast one-hybrid assays confirmed that the Lc protein could combine with the promoter region of *C2* and directly regulate the anthocyanin biosynthesis in the pericarp. These results may provide a new sight to uncover the module and hub genes related to anthocyanins biosynthesis in plants.

## Introduction

Anthocyanins are the visual pigments that present most colors, including black, red, purple, and blue in plants (Paulsmeyer et al., 2017). Some of them take on colorful flowers and function as signals for the attraction of pollinators (Khoo et al., 2017). Some of them are accumulated in fruits and used as essential nutrients for their beneficial effects, including antioxidant activities, antimicrobial, inhibition of lipid peroxidation, and prevention of diseases (Cuevas Montilla et al., 2011; Petroni et al., 2014). Otherwise, anthocyanins play a vital role in protecting the plant from biologically and phenotypically damage such as photodamage and herbivory (Paulsmeyer et al., 2017).

In plants, anthocyanins are biosynthesized *via* the flavonoid metabolism pathway. It is derived from flavonol with the basic structure of flavylium ion. Until now, many kinds of anthocyanins and their derivatives have been identified in natural plants. The most common types are delphinidin, cyaniding, pelargonidin, peonidin, malvidin, and petunidin, which constitute distinct combinations and present various kinds of colors with different concentrations (Khoo et al., 2017). Though the composition of anthocyanins is complex, only several structural genes constitute the main biosynthesis pathway in plants, such as *dihydroflavonol 4-reductase* (*DFR*), *flavanone 3-hydroxylase* (*F3H*), *chalcone synthase* (*CHS*), and so on (Yang et al., 2015). In addition, some transcription factor family genes, including *bHLH*, *MYB*, *WD40*, and *WRKY*, were involved in the anthocyanins biosynthesis (Amato et al., 2017). For example, an MYB transcription factor gene, *BoPAP1* is required for anthocyanin biosynthesis for purple kale (Zhang et al., 2012). A *bHLH* gene, *NnTT8*, functions as *AtTT8* in regulating anthocyanin and proanthocyanin biosynthesis (Deng et al., 2021). A WD40 repeat protein could trigger the biosynthesis of flavan-3-ols by interacting with MYB and bHLH transcription factors in *Camellia sinensis* (Liu et al., 2018). And the ectopic expression of CsWD40 in tobacco enhances anthocyanins biosynthesis in transgenic petals (Liu et al., 2018).

In maize, its stalk, cob, leaf, root, and seed could synthesize and accumulate anthocyanins. Nevertheless, most researchers mainly focus on the anthocyanins in the seed for its edible use. The previous studies reveal that anthocyanins are naturally accumulated in the pericarp and aleurone layer of the seed (Salinas Moreno et al., 2005). Based on the color difference, the seeds with black, red, purple, blue, pink, and bronze pigment were isolated (Paulsmeyer et al., 2017). Further analysis of anthocyanins in the kernels demonstrated that anthocyanin profiles are almost identical, but the contents differ (Salinas Moreno et al., 2005). For example, the content of anthocyanins and total phenolic in red maize were about 5- and 2.5-fold that of blue maize (Ortega-Regules et al., 2012). And the structural genes related anthocyanins biosynthesis in maize were identified, such as *ZmCHS* (*c2*), *ZmCHI* (*chi*), *ZmF3H* (*fht1*), *ZmF3’H* (*pr1*), *ZmDFR* (*a1*), *ZmANS* (*a2*), *ZmUFGT* (*bz1*), and *ZmGST* (*bz2*). Transcription factors including *ZmMYB* (*c1*, *p1*, *pl*), *ZmbHLH* (*Sn*, *r1*, *b1*), and *ZmWD40* (*pac1*) were proved to be the regulatory genes in anthocyanins biosynthesis (Li et al., 2019).

Most previous studies paid more attention to single or several genes associated with maize anthocyanins biosynthesis. In the experiment, the dynamic comparative transcriptome sequence and weighted gene co-expression network analysis (WGCNA) were conducted, and the key modules and hub genes correlated with anthocyanins biosynthesis were analyzed. These results would supplement more hints to understanding the molecular mechanism of anthocyanins biosynthesis and accumulations in maize seeds.

## Materials and methods

### Plant material

The corn inbred line W039 with a black pericarp and an inbred line W042 with a transparent pericarp were isolated from the F7 generations of the hybrid combination W024 and W133 in the farm of Tobacco Research Institute, Anhui Academy of Agricultural Sciences (Fengyang, China). The plants were cultivated on the farm in 2019. After pollination, the pericarps were collected at 15, 20, and 25 days after pollination (DAP). All pericarps were frozen and stored at -70°C refrigerator for the following experiments.

### Microscopic observation and content determination of anthocyanins

The pericarps were separated from the mature seed. Then it was put on a glass slide using tweezers. With a digital camera, the Leica CTR6000 (Germany) was used to observe and document the microstructure. To determine the content of anthocyanins, the mature seeds were ground into powder. Then, 0.1 g samples were collected and resolved in 1 ml of 5% (v/v) formic acid solution by vortex. After ultrasonic vibration at 25°C for 30 min, the supernatants were collected by centrifugation at 6000×g for 10 min. The absorbance was recorded at 530 nm using a microplate reader (Multiskan SkyHigh, Thermo Fisher, USA). The cyanidin 3-sophoroside chloride was selected as standards to calculate the content of anthocyanins.

### The cDNA library construction and sequencing

The RNAprep Pure Plant Kit (Tiangen, China) was employed to extract total RNA from 200 mg pericarp. Three biological replicates were created by independently extracting the RNA three times. The cDNA library construction and RNA-seq were conducted with the help of Beijing Novogene Biological Information Technology Co. Ltd. (Beijing, China) (http://www.novogene.cn/). The RNA-seq was conducted on an Illumina Hiseq 2500 platform with 200bp paired-end reads.

### Data filtering and assembly

The low-quality reads, duplicated sequences, poly-N (reads with unknown nucleotides), and adaptor sequences were removed. The clean reads were mapped to the maize B73 genome (AGPv4) (ftp://ftp.ncbi.nlm.nih.gov/genomes/genbank/plant/Zea_mays/latest_assembly_versions/GCA_000005005.6_B73_RefGen_v4) (Schnable et al., 2009). The TopHat 2 tool was used to conduct the final transcriptome assembly as described by Kim et al. (2013). The fragments per kilobase million mapped reads (FPKM) were calculated based on the method described by Trapnell et al. (2010). All sequence data were stored in the GenBank Short Read Archive and could be accessible with the accession number PRJNA849728.

The genes with low FPKM values (FPKM< 1) were removed first to obtain the candidate genes. Then, the R package (R 4.1.2) (https://cran.r-projet.org/bin/windows/base/) and RStudio Desktop (2022.02.1-461) (https://www.rstudio.com/products/rstudio/download/) were downloaded and installed. The candidate genes’ variance between different samples was calculated using the var function based on the R language server. At last, the top 32% of genes with a maximum level of variance were collected for the following analysis using the quantile function.

### WGCNA analysis

The WGCNA analysis was performed using the method described by Langfelder and Horvath (2008). In detail, the pick-Soft Threshold function command was executed to evaluate the distribution of the quantity matrix and confirm the appropriate power value. Then the adjacency matrix and topological matrix were created based on the calculation of Pearson’s correlations among all genes. Accordingly, the one-step network construction and module detection were conducted to classify the gene modules by marking modules with different colors. The automatic merging function combined the similar modules and obtained the ultimate modules. To get the key module correlated to the anthocyanin biosynthesis in the pericarp, the pigmented pericarp trait value of W039 was specified as “1”, and the transparent pericarp trait value of W042 was set as “0”. The cor order was used to identify the key module related to anthocyanins biosynthesis based on the P-value of the Module-trait relationship. The hub genes were identified based on gene annotation, expression pattern, and gene network analysis. The gene network was displayed using Cytoscape v.3.9.1.

### Metabolism pathway analysis of genes in the key module

To identify the genes associated with the flavonoid biosynthesis pathway, the cDNA sequences of genes in the key module were uploaded to Mercator V3.6 online tools (https://www.plabipd.de/portal/mercator-sequence-annotation) to get root file. The log function calculates the Log Base 2 of the FPKM value. Then, the average values at 15, 20, and 25 DAP were obtained for every gene. The log of the fold changes between W039 and W042 was calculated to present blocks with different colors in mapped target pathways using MapMan software (Version 3.6.0RC1). The MeV software (Version 4.9.0) displayed the genes’ expression pattern. The cluster heatmap was generated using Manhattan Distance with average linkage clustering.

### Yeast one-hybrid experiments

For the yeast one-hybrid experiments, the promoters containing cis-acting elements of *ZmDFR* and *ZmC2* were cloned into the pAbAi vector to construct the pAbAi-baits including proC21-pAbAi, proC22-pAbAi, and proDFR-pABAi (**Table S3**). Then, they were all transformed into *Saccharomyces cerevisiae* strain Y1HGold, respectively. The cDNA pool with flanking end sequences of transcription factor *ZmLc* and the prey vector pGADT7 vector were separately co-transformed into Y1HGolds containing pAbAi-baits. At last, the transformed cells were cultured in a Synthetic Defined (SD) medium without leucine at 28°C for three days.

## Results

### Phenotypic analysis of anthocyanins in pericarps of two inbred lines

**Figure 1:**
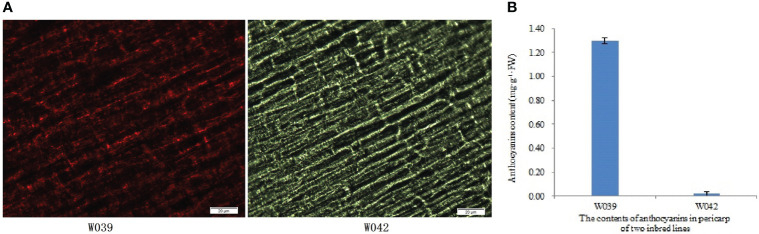
Phenotypic analysis and anthocyanins contents in pericarps of inbred lines W039 and W042. The letter **(A)** indicated the phenotypic observation results. The letter **(B)** showed the anthocyanins content in the pericarps of inbred lines W039 and W042.

In the experiment, two inbred lines, W039 and W042, were obtained. In contrast with the transparent pericarp of W042, the W039 presented dark purple and contained higher anthocyanins content in its pericarp (**Figure 1A, B**).

### Summary of transcriptome data

The pericarps from the seeds of two inbred lines at 15, 20, and 25 DAP were used to construct cDNA libraries. The RNA-seq result showed that 110.01 million raw reads were obtained for six samples, with three biological replicates for each (**Table 1**). After removing low-quality reads, 110.01 million clean reads with 161.94 Gb clean bases were isolated, for which the Q30 value was > 91.67%, and the range content was 53.85-57.60%. The 84.21-86.39% of the clean reads were mapped to the reference genome. The ratios of multiple mapped reads were from 1.85% to 3.01%. The proportions of uniquely mapped reads were over 82%. As a result, 51407 genes were eventually identified for the subsequent analysis (**Table S1**).

**Table 1:** Summary of transcriptome analysis results for pericarps of inbred lines W039 and W056.

Sample name	Raw reads	Clean reads	Clean bases	Total mapped	Multiple mapped	Uniquely mapped	Q30(%)	GC content(%)
W039_15_1	48585334	47999546	7.2G	41154497 (85.74%)	1011935 (2.11%)	40142562 (83.63%)	93.64	54.82
W039_15_2	67008684	64869236	9.73G	54814867 (84.5%)	1438752 (2.22%)	53376115 (82.28%)	92.13	53.97
W039_15_3	60963954	59755838	8.96G	50317753 (84.21%)	1320670 (2.21%)	48997083 (82%)	91.85	53.85
W039_20_1	65176974	64444896	9.67G	56003088 (86.9%)	1662753 (2.58%)	54340335 (84.32%)	93.41	55.67
W039_20_2	52146488	50721608	7.61G	43517380 (85.8%)	1299347 (2.56%)	42218033 (83.23%)	91.97	54.65
W039_20_3	67864960	66755228	10.01G	57174724 (85.65%)	1773203 (2.66%)	55401521 (82.99%)	91.67	54.73
W039_25_1	66644924	65827326	9.87G	57397294 (87.19%)	1880536 (2.86%)	55516758 (84.34%)	93.3	55.2
W039_25_2	65211164	63887864	9.58G	54878176 (85.9%)	1922376 (3.01%)	52955800 (82.89%)	91.58	54.74
W039_25_3	70277630	68506688	10.28G	59214311 (86.44%)	2050638 (2.99%)	57163673 (83.44%)	92.35	54.63
W042_15_1	61469074	60576648	9.09G	52160744 (86.11%)	1289809 (2.13%)	50870935 (83.98%)	93.29	56.43
W042_15_2	57968750	56826576	8.52G	48272653 (84.95%)	1159840 (2.04%)	47112813 (82.91%)	91.86	55.51
W042_15_3	52642966	51572898	7.74G	43748566 (84.83%)	1053974 (2.04%)	42694592 (82.78%)	91.71	55.57
W042_20_1	69802806	68953318	10.34G	58900113 (85.42%)	1378541 (2%)	57521572 (83.42%)	93	57.04
W042_20_2	58461320	57356802	8.6G	48392310 (84.37%)	1121819 (1.96%)	47270491 (82.41%)	91.79	56.13
W042_20_3	56081102	55068998	8.26G	46517208 (84.47%)	1067570 (1.94%)	45449638 (82.53%)	91.7	55.82
W042_25_1	64582936	63662190	9.55G	55000533 (86.39%)	1272361 (2%)	53728172 (84.4%)	93.46	57.6
W042_25_2	57832982	56665698	8.5G	48261687 (85.17%)	1047178 (1.85%)	47214509 (83.32%)	92.06	56.69
W042_25_3	57348180	56231880	8.43G	47898186 (85.18%)	1147817 (2.04%)	46750369 (83.14%)	91.98	56.51

### Isolation of candidate genes for WGCNA

The R package was used for quantitative analysis to acquire the possible candidate genes and expression patterns. The var function was executed to calculate all genes’ variance between different samples. The top 32% of genes with a maximum level of variance were collected for the following analysis using quantile function. Finally, 2057 candidate genes were acquired for the subsequent analysis (**Table S2**).

### Classification of modules for candidate genes

**Figure 2:**
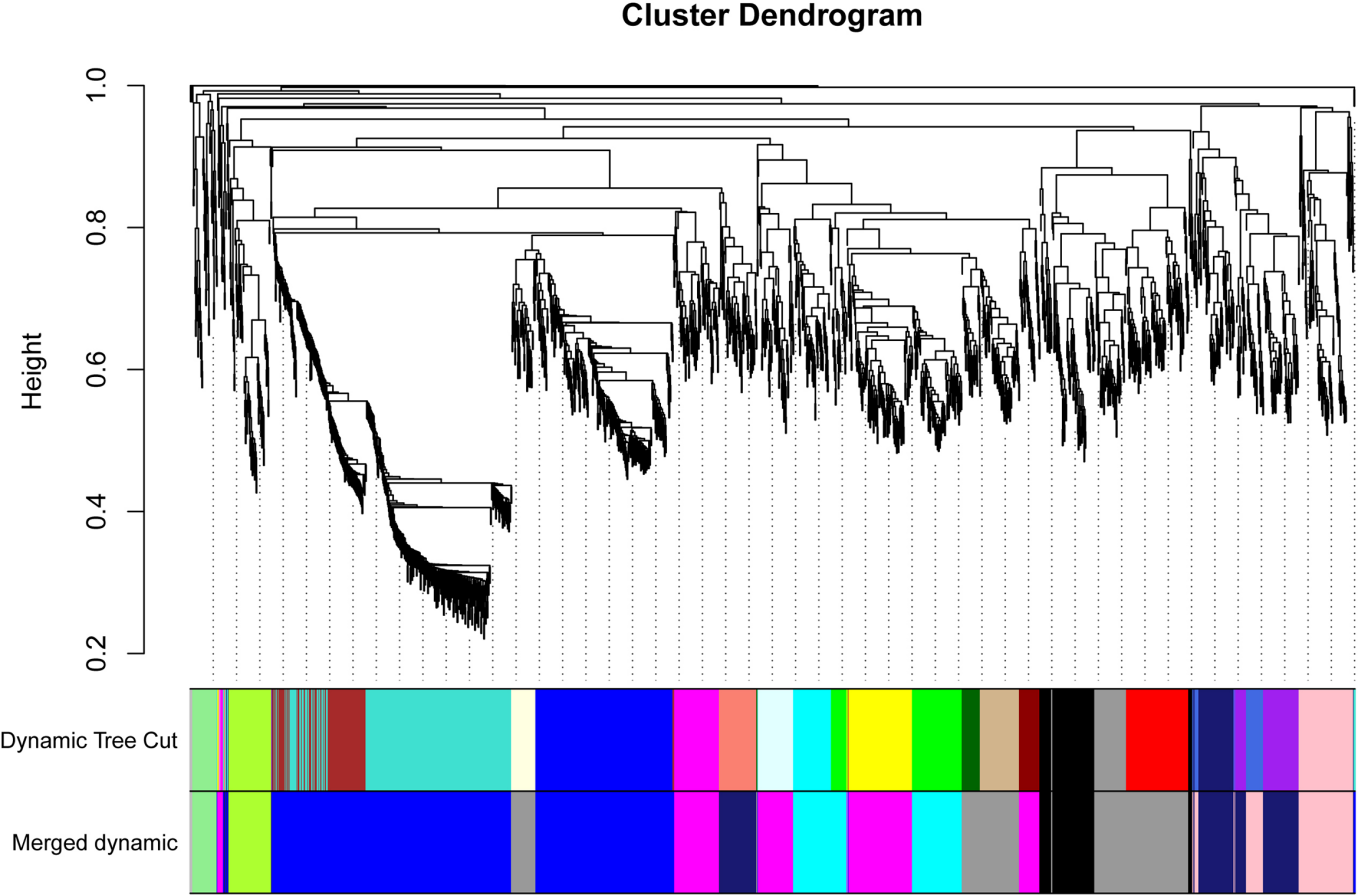
Dendrogram of all 2057 candidate genes clustered based on a dissimilarity measure (1-TOM). The minModuleSize was 30. The MEDissThres was set as 0.2. Different colors displayed the different modules. Each leaf in the tree represented one gene. The major tree branches are originally composed of 23 modules and eventually merged into 10 modules. Each color represented one module.

The WGCNA was conducted to uncover key modules and hub genes using 2057 candidate genes. Based on the soft-thresholding power of 14, the weighted adjacency matrix was created and then transformed into a topological overlap matrix (TOM). All candidate genes were clustered into different modules with similar expression profiles according to the TOM-based dissimilarity measure. The Dynamic Tree Cut with 30 of minModuleSize resulted that 2057 candidate genes being grouped into 23 modules. The eigengenes and dissimilarity of module eigengenes were calculated to cluster similar modules. Using the merging function, 23 modules were eventually combined into ten modules. Among these modules, black and blue modules contained 102 and 678 genes, respectively. The Cyan and greenyellow modules have 183 and 75 genes individually. The grey60 module and lightgreen separately include 314 and 43 genes. The magenta, midnightblue and pink modules were composed of 301, 219, and 137 genes, respectively (**Figure 2**).

### Identification of key modules related to anthocyanins biosynthesis

**Figure 3:**
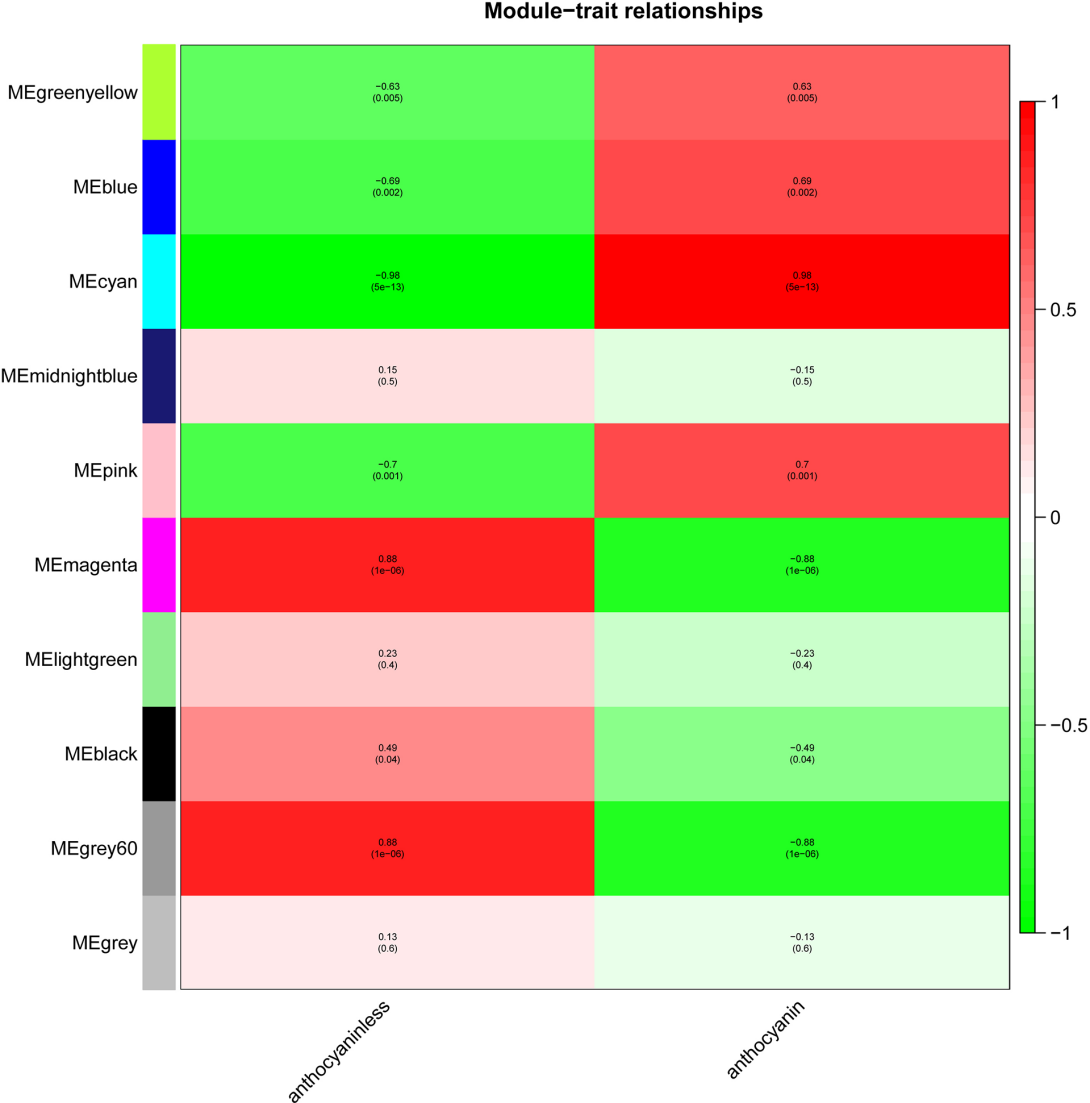
The module-trait relationship between transparent and anthocyanins biosynthesis pericarps. The anthocyanin indicated the pericarp with anthocyanin biosynthesis and accumulation. The anthocyaninless represented the transparent pericarp.

**Figure 4:**
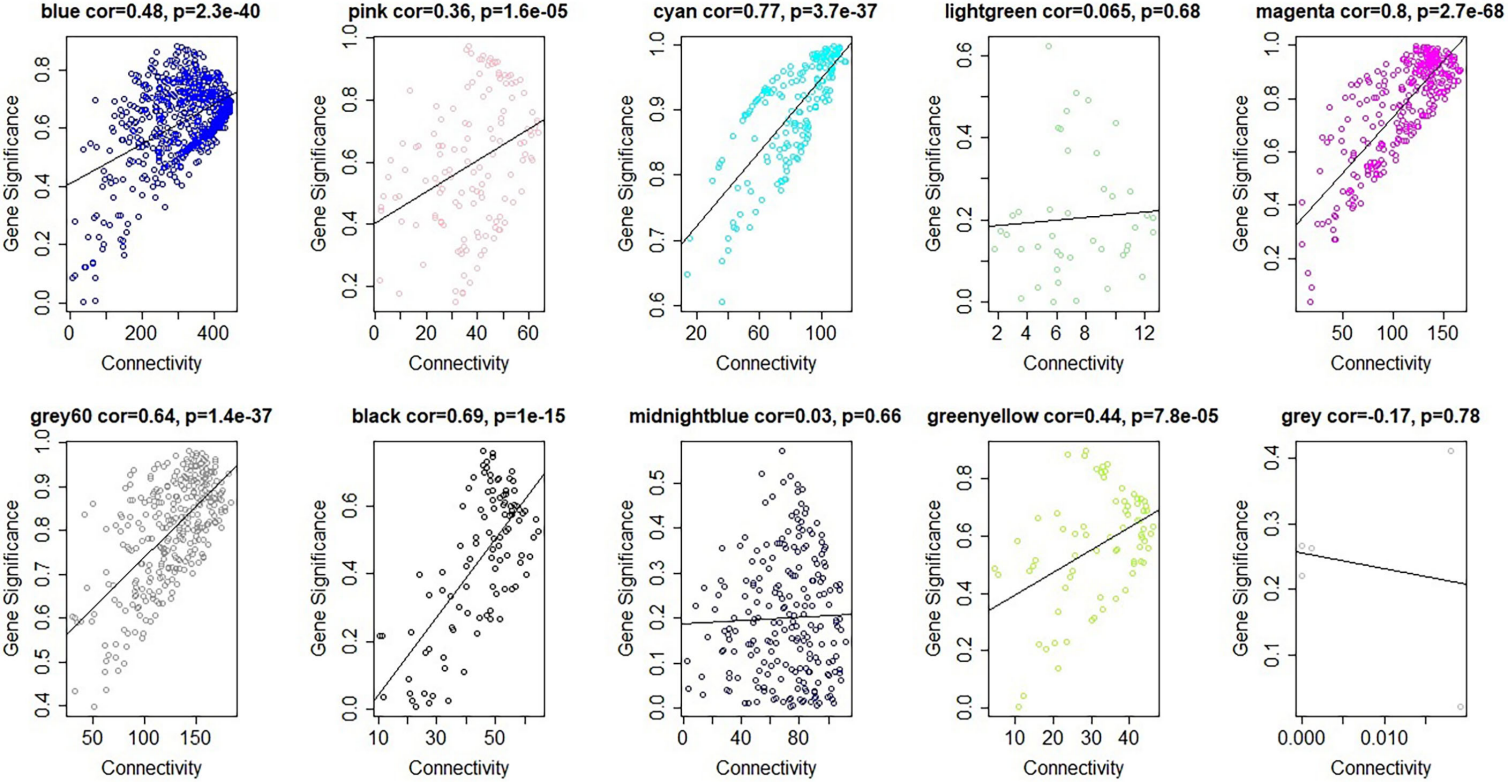
Gene significance vs intramodular connectivity plotted for ten modules. The y-axis indicated the Gene significance, the x-axis displayed the intramodular connectivity. The cyan and the magenta module showed that intramodular hub genes tend to have high gene significance.

**Figure 5:**
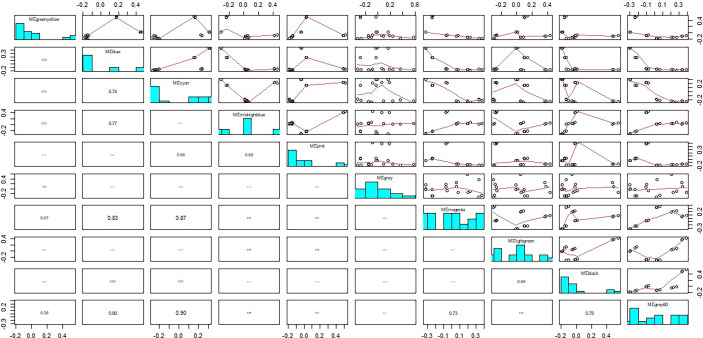
The relationship between module eigengenes. The numbers in column indicated correlation ratios between different modules.

**Figure 6:**
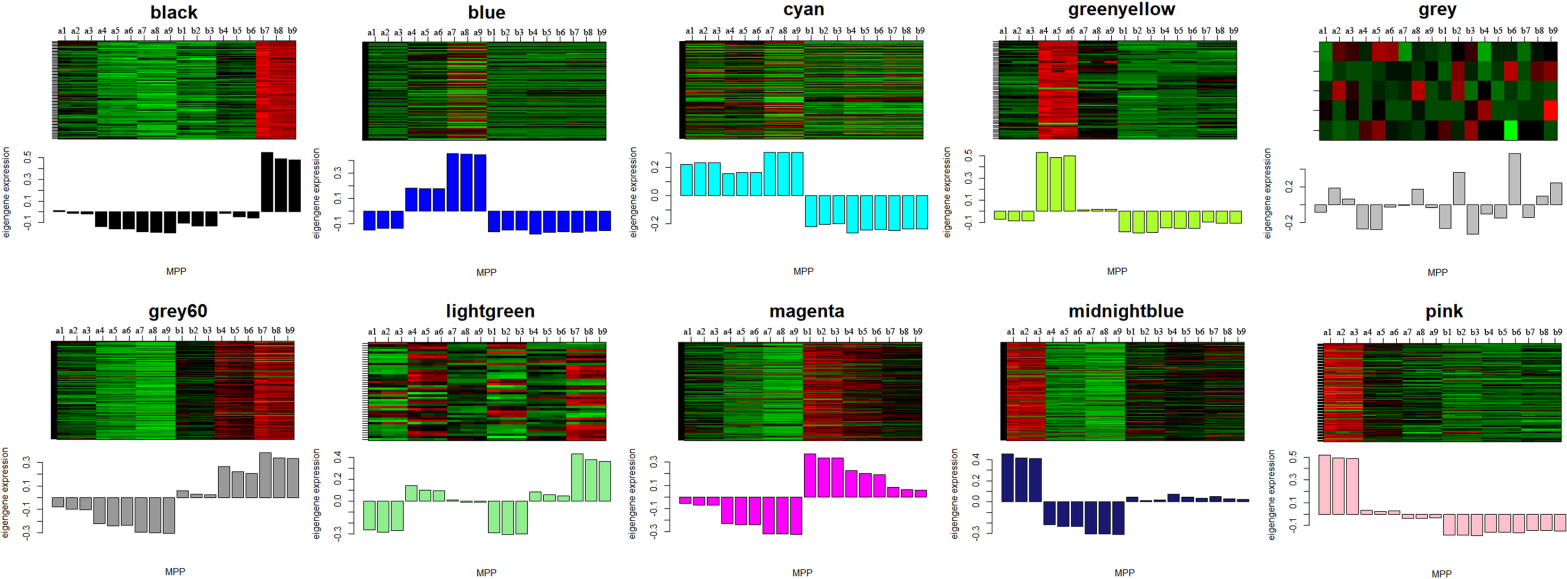
The eigengenes expression patterns and gene expression heatmap across samples for 10 identified modules. In heatmap, the letter a indicated W039, letter b represent W042. The numbers 1, 2 and 3 meant three biological repeats at the 15 DAP stage, 4, 5 and 6 showed three biological repeats at the 20 DAP stage, 7, 8 and 9 represented three biological repeats at the 25 DAP stage.

The module-trait relationships were analyzed using the cor function to obtain the key module and hub genes related to the anthocyanins biosynthesis in the pericarp. The pigmented pericarp trait value of W039 was specified as “1”, and the transparent pericarp trait value of W042 was set as “0”. Based on the Pearson correlation between the trait and transcriptome data, the cyan module was verified to be the key module with the highest correlation value of 0.98 (**Figure 3**). The P-value between the trait and both the magenta and grey60 modules accounted for 0.88. To confirm the above result, the Gene Significance (GS) was calculated to exhibit the associations of individual genes (**Figure 4**). The module membership (MM) was used to display the correlation between the gene expression profile and module eigengene. Then the intramodular analysis was conducted using the GS and MM measures. The result showed that ten modules showed different correlations with the trait. Among all modules, the cyan and magenta modules have a high correlation ratio with anthocyanins biosynthesis (**Figure 4**). Relationship analysis between module eigengenes demonstrated that the cyan module was closely related to the grey60 module and magenta module (**Figure 5**). Otherwise, the eigengene expression pattern and the module expression heatmap were plotted using the plotMat function. As is shown in **Figure 6**, ten modules exhibited distinct eigengene expression patterns and module expression heatmap. Only three modules, cyan, grey60, and magenta, were in accord with the samples at the different development stages. In contrast with grey60 and magenta, the eigengene was positively expressed in pigmented pericarp and negatively expressed in transparent pericarp, which was in accord with the anthocyanins biosynthesis in the tissue. These results confirmed that the cyan module was the key module, which was involved in anthocyanins biosynthesis.

### Identification of hub genes participated in anthocyanins biosynthesis

**Figure 7:**
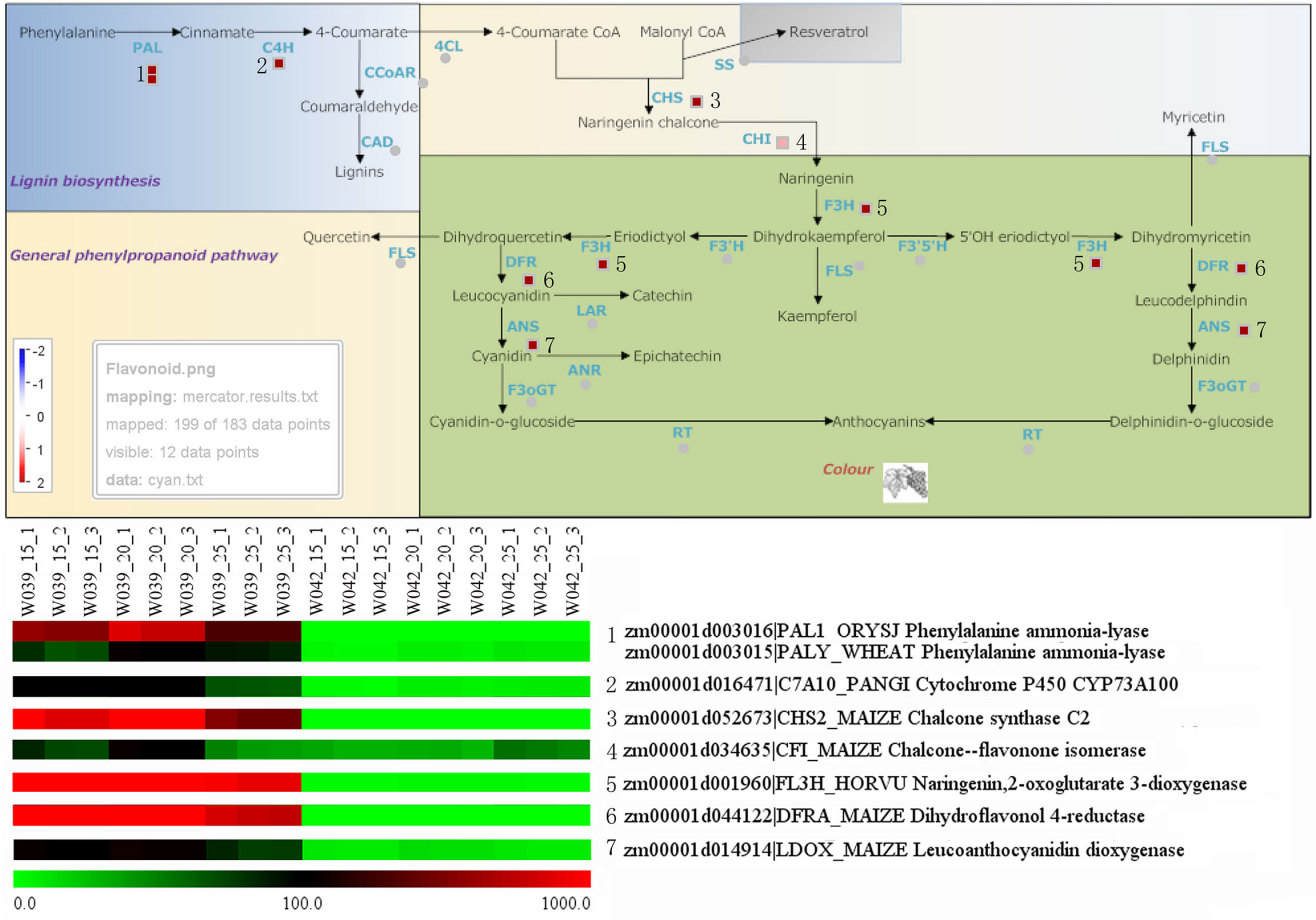
Identification of structural genes related to anthocyanins biosynthesis. The little squares in the pathway represented the LogFC values of W039 vs W042 which were calculated using the average values at 15, 20, and 25 DAP. The listed heatmap exhibited the FPKM value for each gene.

**Figure 8:**
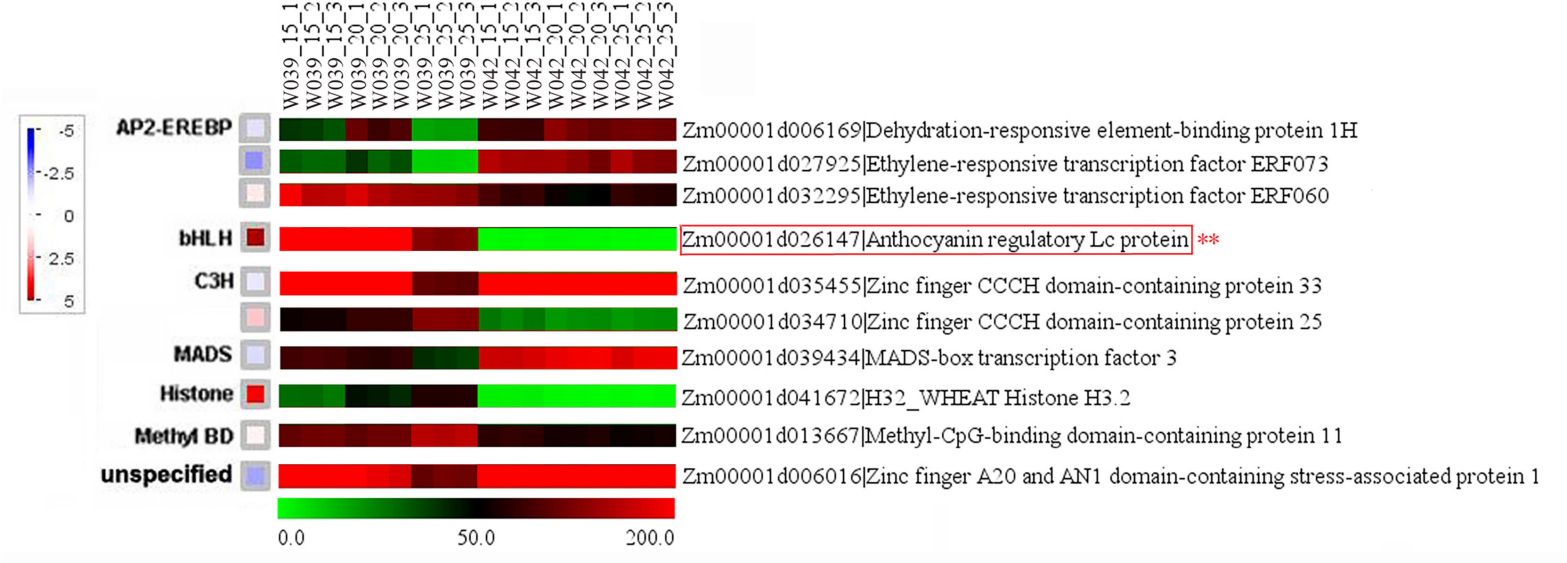
Identification of transcription factors related to anthocyanins biosynthesis. The little squares represented the LogFC values of W039 vs W042 which were calculated using the average values at 15, 20, and 25 DAP. The listed heatmap displayed the FPKM value for each gene..

To identify the hub genes related to anthocyanins biosynthesis, all genes in the cyan module were mapped to the flavonoid biosynthesis pathway using MapMan software. The result showed that eight genes were distributed in the flavonoid biosynthesis pathway and functioned in different sequential steps of the biological process (**Figure 7**). For example, two genes, *Zm00001d003106* and *Zm00001d003015*, were annotated as *Phenylalanine ammonia-lyase* (*PAL*). *Zm00001d016471* functioned as *Cytochrome P450* (*CYP*). *Zm00001d052673* was the *Chalcone synthase* (*CHS*) *C2*, the key gene in the flavonoid biosynthesis pathway. *Zm00001d034625* was marked with *Chalcone flavanone isomerase* (*CHI*). *Zm00001d001960* was identified as *Naringenin, 2-oxoglutarate 3-dioxygenase*. *Zm00001d044122* and *Zm00001d014914* were commented as *Dihydroflavonol 4-reductase* (*DFR*) and *Leucoanthocyanidin dioxygenase* (*LDOX*), respectively. Moreover, ten transcription factors were isolated from the cyan module, including three members of the DREB subfamily A-1 of the ERF/AP2 transcription factor family (Zm00001d006169, Zm00001d027925, and Zm00001d032295), one bHLH transcription factor (Zm00001d026147), two zinc finger (CCCH-type) family proteins (Zm00001d035455 and Zm00001d034710), one MADS-box transcription factor 3 (Zm00001d039434), one histone superfamily protein (Zm00001d041672), one protein containing methyl-CpG-binding domain (Zm00001d013667), and one multiple stress-responsive zinc-finger protein (Zm00001d006016) (**Figure 8**). Among those transcription factors, the bHLH transcription factor Zm00001d026147 was annotated as anthocyanin regulatory Lc protein, which was confirmed to directly participate in the regulation of anthocyanins biosynthesis.

### Verification of hub genes related to anthocyanins biosynthesis

**Figure 9:**
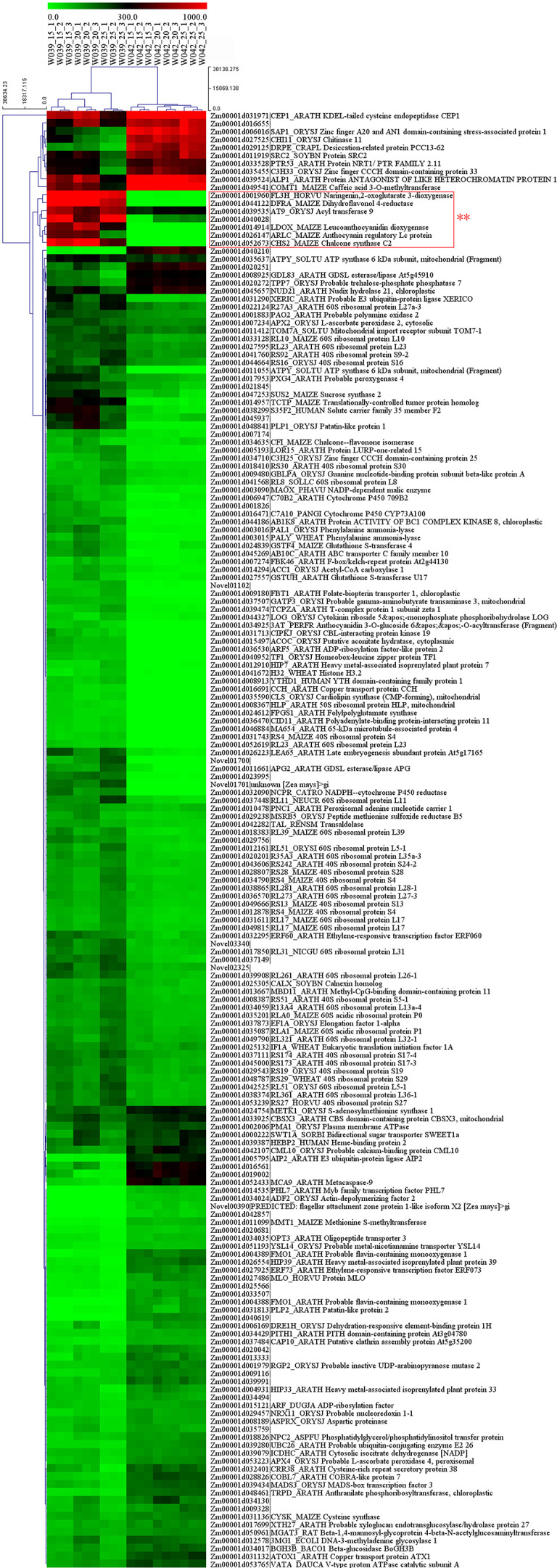
The expression pattern of 183 genes in cyan module. The heatmap represents the FPKM values from RNA sequencing data. The genes in the red square frame marked with double asterisks were categorized to one group and mainly participated in the anthocyanins biosynthesis.

**Figure 10:**
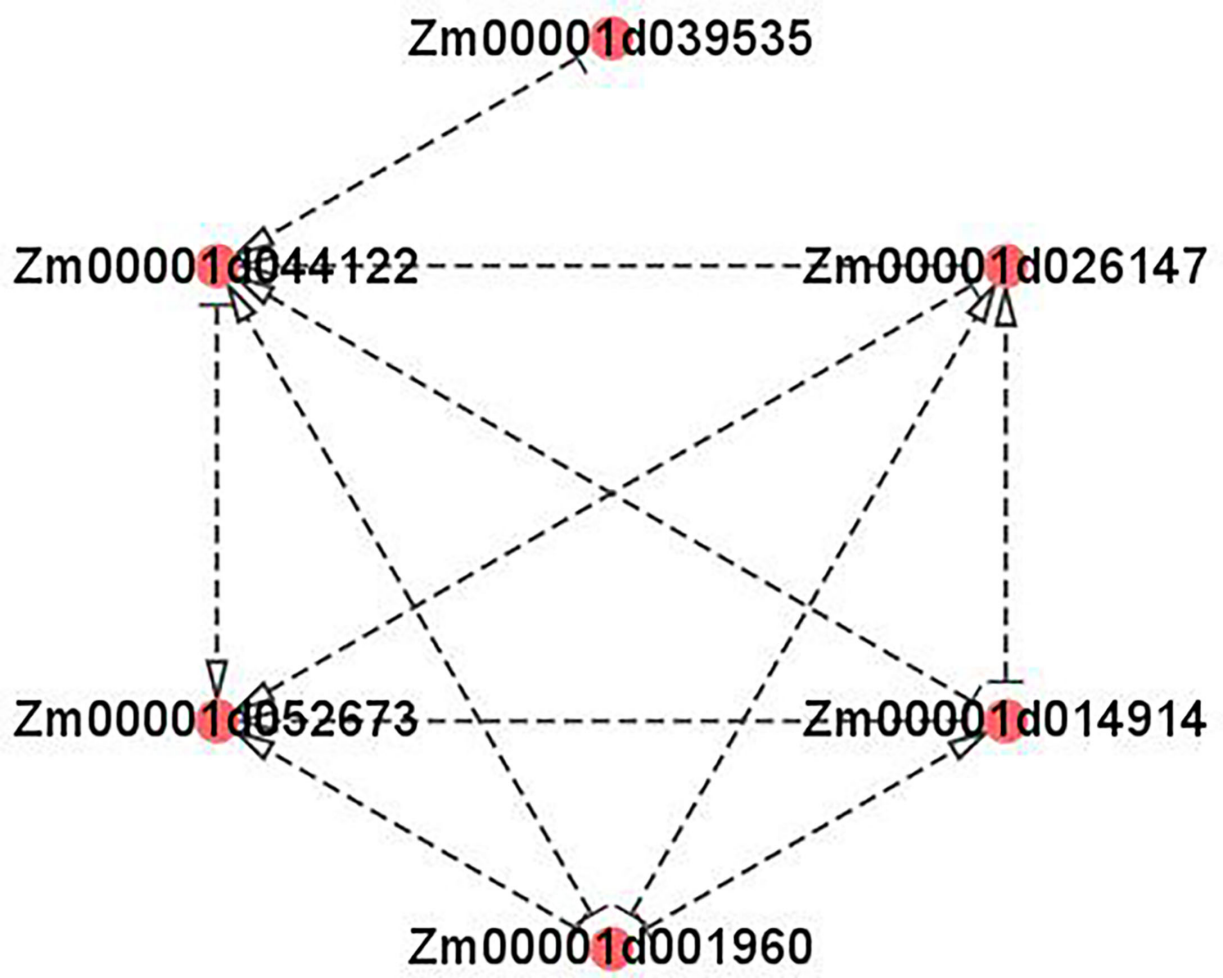
Visualization of co-expression gene network of hub genes by Cytoscape. The arrows pointed the target genes. And the other side of the arrows are the source genes. *Zm00001d039535*: *Acyl transferase 9* (*AcT9*); *Zm00001d044122*: *Dihydroflavonol 4-reductase* (*DFR)*; *Zm00001d052673*: *Chalcone synthase* (*CHS) C2*; *Zm00001d001960*: *Naringenin, 2-oxoglutarate 3-dioxygenase*; *Zm00001d014914: Leucoanthocyanidin dioxygenase* (*LDOX); Zm00001d026147*: *Lc*.

**Figure 11:**
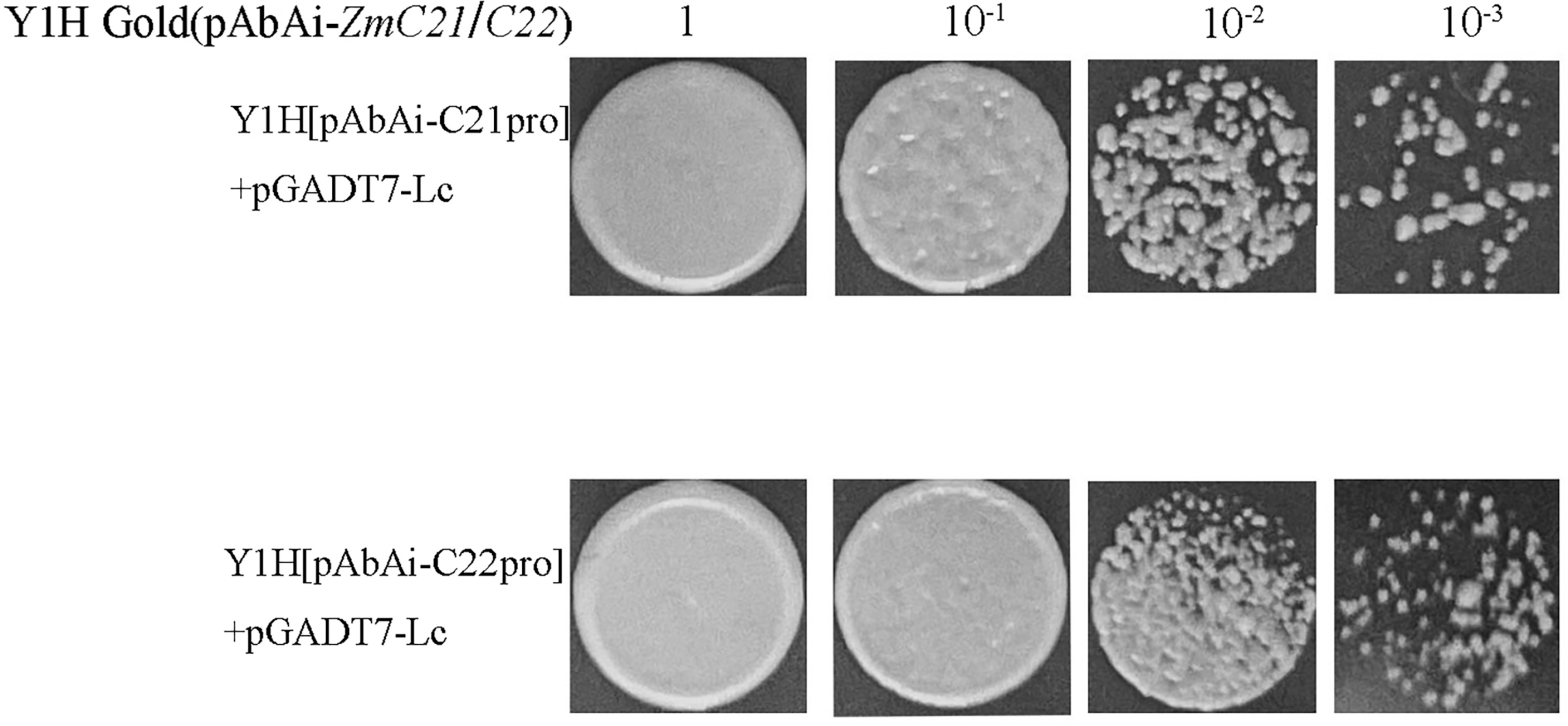
The yeast one-hybrid assays of *in vivo* interaction between *ZmLc* and the target promoter of *ZmCHS*. *ZmC21* and *ZmC22* represented the different region and sequences of the promoter for *ZmCHS*.

As is shown in **Figure 9**, the cluster heatmap was generated to display the genes’ expression matrices in a cyan module using MeV software. The result showed that *Naringenin, 2-oxoglutarate 3-dioxygenase* (*Zm00001d001960*), *Dihydroflavonol 4-reductase* (*DFR)* (*Zm00001d044122*), *Acyl transferase 9* (*AcT9*) (*Zm00001d039535*), *Zm00001d040028* (unknown), *Leucoanthocyanidin dioxygenase* (*LDOX)* (*Zm00001d014914*), *Lc* (*Zm00001d026147*), and *Chalcone synthase* (*CHS) C2* (*Zm00001d052673*) were categorized to one group. Among those genes, *Naringenin, 2-oxoglutarate 3-dioxygenase* (*Zm00001d001960*), *Dihydroflavonol 4-reductase* (*DFR)* (*Zm00001d044122*), *Leucoanthocyanidin dioxygenase* (*LDOX)* (*Zm00001d014914*), *Lc* (*Zm00001d026147*), and *Chalcone synthase* (*CHS) C2* (*Zm00001d052673*) were all participated in the anthocyanins biosynthesis. Gene network analysis revealed that the transcription factor *Zm00001d026147* act on the genes *Zm00001d052673* and *Zm00001d044122* (**Figure 10**). The yeast one-hybrid assays demonstrated that the *Lc* gene (*Zm00001d026147*) could combine with the promoter region of *C2* (*Zm00001d052673*), which directly regulated the anthocyanin biosynthesis in the pericarp (**Figure 11**).

## Discussion

### The application of WGCNA in identification of modules and hub genes

Recently, WGCNA has been widely used to find modules of highly correlated genes and relate modules to one another and external sample traits (Langfelder and Horvath, 2008). It has been proved to be an effective tool for identifying key modules and hub genes correlated with target traits. It was successfully applied in various biological experiments, especially in mammals disease studies, including identifying the mechanism of breast cancer, revealing prognostic genes among diffferent cancer types, and uncovering key drivers of acute peanut allergic reactions (Guo et al., 2017; Watson et al., 2017; Ricketts et al., 2018). In plants, the WGCNA has been conducted to isolate the modules and hub genes related to seed development, metabolism, and stress responses (Du et al., 2017; Garg et al., 2017; Ma et al., 2017). This study obtained 18 samples’ transcriptomes at 10, 15, and 20 DAP, respectively. Using the quantile function of the R language, 2057 genes were filtrated out from 51407 genes based on FPKM values. Eventually, seven genes in the cyan module were mapped to the anthocyanins biosynthesis pathway, which participated in anthocyanins biosynthesis in the pericarp.

### Structural genes and transcription factors related to anthocyanins biosynthesis in plants

Anthocyanin biosynthesis requires the coordinated expression of structural and regulatory genes (Ma et al., 2009). Regulatory genes, including *bHLH*, *R2R3-MYB*, *WRKW*, and *WD40*, have been widely identified in *Petunia hybrida*, apples, pears, and other plants (Li, 2014; Zhang et al., 2019). It is reported that R2R3-MYB proteins MYB11, MYB12, and MYB111 could trigger the expressions of the genes *CHS*, *CHI*, *F3H*, and *FLS1* (Li, 2014). In apple, an *MdbHLH2* gene was able to bind to the promoters’ sequences of the genes *MdDFR*, *MdUFGT*, and *MdMYB*. It could enhance anthocyanin accumulation induced by low temperature in fruit (Xie et al., 2012). Otherwise, a WD40 protein MdTTG1 could interact with bHLH transcription factors and indirectly regulate the anthocyanin biosynthesis (An et al., 2012). In red pear, the transcription factors PyMYB10, PybHLH, and PyWD40 formed a ternary complex to regulate anthocyanin biosynthesis in the peel under the sunlight (Cui et al., 2021). In kiwifruit, two transcription factors, MYBC1 and WRKY44, triggered the expression of *F3′H* and *F3′5′H* genes by activating their promoters (Peng et al., 2020). Moreover, AcMYB123 and AcbHLH42 could bind to promoters of *AcANS* and *AcF3GT1* and activate their expressions. The co-expression of two genes is a prerequisite for anthocyanin production in *Actinidia c*ultivars (Wang et al., 2019). In chrysanthemums, *CmbHLH2* could bind to the *CmDFR* promoter and trigger anthocyanin biosynthesis (Xiang et al., 2015). In addition, a bHLH protein PsGBF could bind to the G-box cis-element of the PsCHS1 promoter and regulate anthocyanins biosynthesis in pea (Qian et al., 2007). In Zea mays, the members of two families’ genes of *R2R3-MYB* and *bHLH* have been isolated, too. The members of the *c1* family, including *c1* and *pl*, were annotated as *MYB* transcription factor genes, while the members of the *r* family, such as *r*, *lc*, *sn* and *b*, belong to bHLH transcription factor genes (Quattrocchio et al., 1998). The overexpression of maize *ZmC1* and *ZmR* genes in wheat initiated anthocyanin biosynthesis (Riaz et al., 2019). Ectopic expression of *Lc* enhanced the accumulation of total anthocyanin contents in tobacco’s calyxes, petals, and filaments. The expressions of anthocyanin biosynthetic genes *NtCHS*, *NtDFR*, *NtF3′H*, and *NtANS* were in accord with the expression profile of the *Lc* gene (Huang et al., 2016). In the experiment, several structure genes, including *Phenylalanine ammonia-lyase* (*PAL*), *Chalcone synthase* (*CHS*) *C2*, *Chalcone flavanone isomerase* (*CHI*), *Naringenin, 2-oxoglutarate 3-dioxygenase*, *Dihydroflavonol 4-reductase* (*DFR*), *Leucoanthocyanidin dioxygenase* (*LDOX*), and one regulatory gene *Lc* gene were identified using WGCNA analysis based on dynamic comparative transcriptome sequence. Gene network and yeast one-hybrid verified that the *Lc* gene could bind to the promoter of *C2* and further regulate the anthocyanin biosynthesis (**Figure 11**).

## Conclusion

In the experiment, the transparent and anthocyanins-enriched pericarps at 15, 20, and 25 DAP were used to conduct dynamic comparative transcriptomes analysis. The results output 110.007 million raw reads and 51407 genes’ expression matrix. After filtration, 2057 genes were eventually identified as the candidate genes for weighted gene co-expression network analysis. As a results, ten modules were obtained and the cyan module containing 183 genes was the key module correlated with anthocyanins biosynthesis. Among 183 genes, four structural genes including *Naringenin, 2-oxoglutarate 3-dioxygenase*, *Dihydroflavonol 4-reductase* (*DFR*), *Leucoanthocyanidin dioxygenase* (*LDOX*), and *Chalcone synthase* (*CHS*) *C2* and a transcription factor *Lc* gene participated in the anthocyanins biosynthesis. And the transcription factor anthocyanin regulatory *Lc* gene could bind to the promoter region of *C2* and directly regulate the anthocyanin biosynthesis in the pericarp. These results may provide a new sight to uncover the module and hub genes related to anthocyanins biosynthesis in plants. And the WGCNA will be the effective tools to identify target genes and its correlated gene network associated with the plant phenotypic trait.

## Data availability statement

The datasets presented in this study can be found in online repositories. The names of the repository/repositories and accession number(s) can be found in the article/**Supplementary Material**.

## Author contributions

TL and CL designed the experiment and performed WGCNA analysis. YW and QD helped with transcriptome analysis. FW and KF helped with phenotypic analysis and yeast one-hybrid experiments. GL, YL, HY, and YZ were responsible for cultivating corns and preparing pericarps. All authors have read and approved the final manuscript. All authors contributed to the article and approved the submitted version.

## Funding

This work was supported by the Key Research and Development Plan of Anhui Province (202104a06020019), the National Natural Science Foundation of China (31601240), the Natural Science Foundation of Anhui Province (2008085MC74), and the Anhui University research project (YJS20210230).

## Conflict of interest

The authors declare that the research was conducted without any commercial or financial relationships construed as a potential conflict of interest.

## Publisher’s note

All claims expressed in this article are solely those of the authors and do not necessarily represent those of their affiliated organizations, or those of the publisher, the editors and the reviewers. Any product that may be evaluated in this article, or claim that may be made by its manufacturer, is not guaranteed or endorsed by the publisher.

## Supplementary material

Supplementary Table 1 All genes identified by transcriptome analysis and their distribution on chromosomes.

Supplementary Table 2 The list of 2057 candidate genes and their belonging module.

Supplementary Table 3 Primers with homologous arms sequences for promoters of target genes.
