# Supplementary material for: Weighted gene co-expression network analysis reveals key module and hub genes associated with the anthocyanin biosynthesis in maize pericarp
Source: Front Plant Sci. 2022 Oct 31;13:1013412. doi: 10.3389/fpls.2022.1013412 (PMC9661197; doi:10.3389/fpls.2022.1013412)
Supplement: Supplementary Table 3 — Primers with homologous arms sequences for promoters of target genes. [file Table_3.docx]

Tables S3 Primers with homologous arms sequences for promoters of target genes

| Name | Sequences |
| --- | --- |
| proC21F | aattcgagctcggtacccgggCCGAGTGCCTCAGACCCAG |
| proC21R | atacagagcacatgcctcgagCACGCACTCGACAAAGAAGGC |
| proC22F | cttgaattcgagctcggtaccGTGTCGTACGTATTCTCTAGGAAAGTAAG |
| proC22R | agcacatgcctcgaggtcgacCCGCTTGACGTAAAACAGTCG |
| proDFRF  proDFRR | cttgaattcgagctcggtaccCTAGCTAGGCGTGTCAATTTATTGA  cttgaattcgagctcggtaccCTAGCTAGGCGTGTCAATTTATTGA |
| proLcF | gccatggaggccagtgaattcATGGCGCTTTCAGCTTCCC |
| proLcR | cagctcgagctcgatggatccTCACCGCTTCCCTATAGCTTTG |
